# LGBTQ+ Students’ Experiences of Misnaming in Swedish Secondary Schools

**DOI:** 10.3390/healthcare14010013

**Published:** 2025-12-20

**Authors:** Paul Horton, Camilla Forsberg, Ben Lohmeyer

**Affiliations:** 1 Department of Behavioural Sciences and Learning, Linköping University, 581 83 Linköping, Sweden; 2 College of Education, Psychology and Social Work, Flinders University, Adelaide, SA 5042, Australia

**Keywords:** school bullying, misnaming, LGBTQ+, transgender, nonbinary, school staff, qualitative

## Abstract

**Background/Objectives:** Bias-based bullying is widely recognised as having a detrimental impact on child and adolescent health. One form of bias-based bullying that is directed at transgender and non-binary students more specifically is misnaming, whereby someone is referred to by their birth name rather than their chosen name. While there have been some studies exploring the experiences of young LGBTQ+ people’s experiences of bias-based bullying, and a number looking at misnaming more specifically, there has been very little research on the issue in the Swedish school context. The aim of this study is to address this lacuna in knowledge by focusing on LGBTQ+ students’ experiences of misnaming in Swedish schools. **Methods:** The findings are based on 21 semi-structured interviews with LGBTQ+ young people aged 15–25 from around Sweden. The interviews were analysed with reflexive thematic analysis and in relation to the concepts of orientations and affect. **Results:** The findings highlight how misnaming negatively impacts the health of those who are subjected to it through disorientation and by triggering negative emotions such as anger, sadness, anxiety, and hurt. The findings also illustrate how the affects of misnaming extend beyond individuals and accumulate as collective emotional experiences that negatively transform schools into sites of personal and social tension. **Conclusions:** The article demonstrates not only the importance of recognising misnaming as a pernicious form of bias-based bullying that negatively impacts the health of students, but also the need for schools to actively review their administrative systems to ensure that misnaming is not institutionalised and perpetuated within schools.

## 1. Introduction

School bullying is widely recognised to be a significant problem globally [1,2]. Recently, following extended discussions between international bullying researchers, a new definition of school bullying was put forward by UNESCO, which emphasises that school bullying involves more than the negative actions of individuals or groups of individuals:

School bullying is a damaging social process that is characterized by an imbalance of power driven by social (societal) and institutional norms. It is often repeated and manifests as unwanted interpersonal behaviour among students or school personnel that causes physical, social, and emotional harm to the targeted individuals or groups, and the wider school community [3] (p. 4).

Highlighting the importance of the social, societal and institutional norms pointed to in the revised definition above, some bullying researchers have focused explicitly on bias-based bullying; a subtype of bullying that targets particularly those who belong, or are perceived to belong, to social categories that tend to be stigmatised in society and institutions like schools [4,5,6]. Of particular relevance for the focus of our study are forms of bias-based bullying used to target students perceived as or identifying as lesbian, gay, bisexual, transgender, queer/questioning, or any other sexual orientation or gender identity (LGBTQ+), such as homophobic, biphobic, and transphobic bullying [7]. International research has found that LGBTQ+ students are at greater risk of being bullied than their heterosexual and cisgender peers and are also at greater risk of being negatively affected by the bullying to which they are subjected [1,8,9,10,11,12].

The Swedish Discrimination Act (Diskrimineringslag) [13] legislates against discrimination and harassment based on discriminatory grounds such as gender, transgender identity, and sexual orientation. However, despite this legislation obligating schools to actively counter discrimination and harassment and to have guidelines and routines in place for its prevention, recent reports highlight that LGBTQ+ students are regularly faced with non-inclusive school environments and subjected to bias-based bullying. Recent surveys from the anti-bullying organisation Friends, for example, have found that transgender identity and sexual orientation are two of the most common grounds for harassment [14] and that 25% of Swedish school students have received sexist or homophobic comments at school [15]. Likewise, a recent report from Save the Children Youth Sweden [16] found that ‘the vast majority’ of the LGBTQI-identifying children with whom they held co-creation groups expressed that it was ‘very common to be treated badly at school because of their identity or perceived identity’ (p. 20). Recent newspaper reports have also suggested that LGBTQ+ hostility and intolerance is on the increase in Swedish schools [17,18].

International research has demonstrated that transgender and non-binary students are commonly subjected to two particular forms of bias-based bullying: misgendering and misnaming [4,19,20,21,22,23,24]. Misgendering is when someone is addressed in ways that do not correctly reflect their gender identity, such as by using the wrong pronoun, while misnaming is when someone is referred to by their birth name rather than the name with which they identify [20,21]. Studies have shown that school staff may be unaware of the prevalence of misgendering and misnaming due to its subtle nature [20] or may perceive such interactions as micro-aggressions that are not adequately serious to follow up in a systematic fashion [21,24]. Some studies have also found that students experience being subjected to misgendering and misnaming by school staff [19,24,25] and by the institutional ‘cistem’ through old names remaining on administrative platforms and student lists [23] (p. 385) [24,25]. Researchers have highlighted that misnaming is an invalidating, offensive, and harmful practice [24] that may be experienced as an attack on the self and one’s agency to decide how one is identified, as the practice forcefully pulls that person’s past back into their present [23].

While there have been numerous studies looking at young LGBTQ+ people’s experiences of bias-based bullying, and misgendering and misnaming more specifically, there has been relatively little research on the issue in the Swedish school context [26,27,28,29]. The aim of this study is to address this lacuna in knowledge about the experiences of LGBTQ+ young people in Swedish schools, and particularly in relation to experiences of misnaming. While misgendering and misnaming may often be used within the same bullying situations, in this study we focus specifically on LGBTQ+ students’ experiences of misnaming in Swedish schools. We use the term misnaming rather than the commonly used term ‘deadnaming’, as despite many of the young people in our study using the terms ‘deadname’ and ‘deadnaming’, some use the terms ‘old name’ and ‘birth name’ instead and are perhaps not comfortable with the ‘killing’ of the name they were assigned at birth [23]. Drawing on the work of Sara Ahmed [30,31,32,33], we utilise the concepts of orientations and affect as means of exploring young LGBTQ+ people’s school experiences of misnaming.

## 2. Theoretical Framework

Sara Ahmed’s concept of orientations is a useful starting point for considering the school experiences of young LGBTQ+ people, because as Ahmed notes, orientations relate to people’s starting points and how they proceed from there [33]. Indeed, orientations help us to understand how school experiences may take very different forms depending on the ways in which schools and school spaces are orientated towards some people more than others, through norms, school practices, and so on. As Ahmed argues, spaces are designed for some people more than others [30]. As we will demonstrate, cisheteronormativity underpins a number of ‘straightening’ [30] (p. 72) and ‘stopping’ [30] (p. 139) devices within schools that are utilised to ensure that people stay ‘on line’ and do not stray ‘off line’, i.e., deviate from the cisheteronormative line advocated and followed by the majority [30] (p. 70). The ways in which schools are orientated determine the extent to which certain students may feel in place, or at home, while others may experience disorientation and hence a sense of being out of place or losing their place [30,31]. As Ahmed points out, disorientation may be experienced violently and might lead to an accumulation of stress, which is experienced as pressure upon the body [30].

Acts of naming and misnaming make their mark and impress upon the body [32]. Studies have found, for example, that when young trans people are addressed through use of their chosen names, they have lower reported rates of depression, suicidal ideation and suicidal behavior [23]. In contrast, when someone’s preferred name is not used when addressing them, they may experience it as their sense of self being attacked and their agency being taken away from them [23]. Acts of misnaming affect not only the person who is misnamed but also the relations between those doing the misnaming and those being misnamed. Rather than emotions being possessed or internal to an individual body [34], a social approach to affect draws attention to the circulation of emotions between bodies and how the social impacts the person and on emotions [35]. Emotions, understood socially rather than individually, work to align some people with certain other people and to distance them from others [36]. Emotions are not just psychological disposition but are about power. Misnaming, therefore, is more than the actions of an individual. It is part of the operations of power in a social space and understanding it thus requires an analysis of the social context—in this case the school.

Ahmed describes the circulation of emotions in a space as an ‘affective economy’, arguing that emotions work like a form of capital [36]. Drawing from Marx, she argues that, in the circulation of emotions between bodies, they accumulate or gain value over time. As a form of capital, emotions are not simply internal states but impact the world. When emotions align certain people within a community, and create distance between others, they contribute to patterns of inclusion and exclusion [33,34]. In schools, emotions flow and collide with one another and contribute to the formation of affective landscapes [34,37]. Exploring these landscapes has provided insight into the emotional discourses and practices underpinning the socio-spatial dynamics of racism in multicultural schools [34] and mapped the affect of microaggressions and microaffirmations on disenfranchised students [37]. In this paper, we employ this framework to elucidate the ‘hammering’ or ‘constant chipping away’ [38] (p. 22) that acts of misnaming perform on transgender and non-binary students at school.

Ahmed employs the analogy of a skin irritation to illustrate affectivity. People are affected by the things and people with whom they come into contact, and as a result affect travels [38,39]. Rather than simply perceiving emotions as things that are held by people (e.g., ‘I am sad’), then, when considering the social aspect of emotions, it is possible to explore how the everyday social environment of students may be affected in different ways by acts of misnaming. As Ahmed eloquently puts it, the ‘skin of the social’ can be understood as ‘a border that feels and that is shaped by the “impressions” left by others’ [30] (p. 9). In a similar way, Ringrose and Rawlings describe how the gendered associations with certain spaces in schools, such as masculinity in a gymnasium, make a mark and leave lasting impressions on the body [40]. As emotions are an embodied experience, these impressions are carried around and end up being experienced as part of the self [38]. They may create connections or distance between different people (e.g., students and teachers) and objects (e.g., students and safe or unsafe spaces). Indeed, as Ahmed points out, emotions not only circulate but also ‘stick’ [32] (p. 4). This is particularly important when thinking about experiences of misnaming in schools, as the feelings evoked by such experiences may affect the ways in which students, staff and school spaces become saturated with affect, and hence become sites with a lingering history of emotional tension [32].

## 3. Materials and Methods

The findings presented in this article draw from a larger qualitative interview study investigating LGBTQ+ young people’s experiences of bullying at school in Sweden. To be included in the study, participants had to be between the ages of 15 and 19, living in Sweden, and to self-identify within the LGBTQ+ spectrum. Those who did not meet these criteria were excluded from the study, although participants were able to choose to be interviewed with friends. This was the case in one interview, as explained below. Various means were used to find and recruit participants for the research, including advertising on social media platforms, emailing LGBTQ+ organisations and associations, and contacting upper secondary schools and youth clubs.

Twenty-one young people were interviewed from all around Sweden. This included young people from rural areas, small towns, and medium to large cities. The interviews were conducted either face-to-face or online, depending on the locations and preferences of the participants. Half of the interviews were conducted online, while half were conducted face-to-face at locations that the participants chose and where they felt comfortable. This included cafes, youth clubs, and a university campus. All of the interviews were conducted individually, except for one group interview, which was conducted with three young people who were friends (one of whom was 25 years old). The interviews were conducted in Swedish and ranged in duration from 42 min to 1 h and 46 min, with the majority averaging roughly one hour. The interviews were semi-structured and based on an interview guide with a broad range of questions, which provided flexibility for allowing the participants adequate space to answer the questions in their own words [41].

The interviews were audio-recorded and transcribed to facilitate analysis. The transcription function in Word was utilised in the transcription process. Similar to other studies exploring the experiences of LGBTQ+ young people through the use of interviewing, e.g., [21,22,23,25], we utilised reflexive thematic analysis to make sense of the interview data [42]. Reflexive thematic analysis was chosen because it offers a flexible data analysis method for identifying, analysing, and interpreting patterns in qualitative data related to, for example, people’s lived experiences and perspectives. In this sense, it is an interpretive approach that facilitates understanding of subjective experiences, feelings, emotions, and so on. In line with reflexive thematic analysis, the interview transcripts were read through multiple times to aid familiarisation and distance from the data. Initial codes were then generated by the first and second authors that captured our ‘analytic take’ on the data [42] (p. 35). The codes were then compiled under themes (such as ‘misnaming’ and ‘misgendering’) that were then reviewed and revised, before being considered in relation to previous research and the theoretical concepts informing the study. During the writing up of this article, excerpts have been selected for further analysis in relation to the focus on experiences of misnaming.

The study is funded by the Swedish Research Council (2022-04513) and ethical approval was sought and approved from the Swedish Ethical Review Authority (Dnr 2023-01518-01) prior to the research being undertaken. All the participants were informed about the focus of the study, that they were free to refrain from answering any questions, to end their participation at any time, that their answers would be treated confidentially, and that they would be anonymised in the writing up of the findings. This information was provided either online or in-person during the recruitment process and participants were reminded of the information at the beginning of their interview. All participants provided consent to participate, either verbally or in writing, prior to being interviewed. All the names used in this study are pseudonyms to protect the identity of the participants.

## 4. Results

All the LGBTQ+ students in our study had experiences of bullying, whether directed at themselves or at fellow LGBTQ+ young people in their schools. This highlights the extent to which their schools were orientated in cisheteronormative ways and how identifying as LGBTQ+ was considered being out of line and out of place [30]. In discussing their experiences, the young people repeatedly raised the issue of misnaming, whether it was done by peers or via the institutionalisation of misnaming in the administrative school system. In presenting our findings, we discuss each of these forms of misnaming in turn, beginning with experiences of misnaming by peers.

### 4.1. Misnaming as an Emotional Trigger

Lo, who was 15 at the time of being interviewed, pointed to the cisheteronormative orientation of his lower secondary school, wherein there was a perception that ‘LGBTQ people are just weird and a bit like that’ and trans students were ‘deadnamed’. Lo provided examples of not only being misnamed, but also having his correct name used in a taunting way by peers. Skylar, who was 18, also pointed to the cisheteronormative orientation of her upper secondary school:

At my school, I think there is a very mean rhetoric and jargon regarding LGBTQ. Especially when it comes to transgender people and non-binary people. It happens a lot that they are, what do you say, undermined I think is a good word, and yeah but ridiculed and stuff and there are a lot of people who don’t respect people’s identities and are generally ignorant about LGBTQ things. Issues and stuff. You sometimes hear, for example, that non-binary people don’t exist. So that’s how it is at my school, specifically for my class too.

The rhetoric and jargon that Sklyar refers to illuminate a specific school- and class-climate that is generally hostile to LGBTQ+ people, and particularly transgender and non-binary people, and positions them as out of line and out of place [30]. Rather than being respected as equal members of the school community, Skylar suggests that LGBTQ+ people, and particularly those who identify as trans or non-binary, are instead ‘undermined’. Through this undermining process, they are negatively distinguished from their peers, ridiculed, and their humanity is chipped away to the extent that they are perceived as no longer existing. While Skylar was, in her words, ‘in the closet’ and had not revealed her transgender identity at school, she explained that her experiences at school had negatively affected her and triggered feelings of ‘anxiety or stress’ about what would happen if she was open about her gender identity at school. These ‘stress points’ served to block her ability to extend herself into the school setting [30] (p. 160). Indeed, Skylar expressed that if she came out, she too would probably be subjected to misgendering and misnaming from her fellow students. In other words, Skylar’s experiences of the cisheteronormativity of her school context had not only left an impression on her personally but also served to shape the skin of the social and hence her relations with her peers by creating distance between them. Furthermore, Skylar’s experience illustrates not only a personal emotional experience, but how emotions circulate, accumulate, and stick—‘a lot of people who don’t respect people’s identities’—and the school context itself was thus saturated with affect and feelings of anxiety and stress.

Matti, who was 16 and had just started upper secondary school, explained that while he was not open about being trans at his lower secondary school, he had officially changed his name and that this had been used by other students to target him. Highlighting the cisheteronormative orientation of the school climate, Matti elaborated on an example that he remembered most vividly. He had been at the school cafeteria and had felt things were getting better, as other students had begun to get used to his new name and had not subjected him to misnaming in a while. He described how after finishing ladling food onto his plate, he passed the ladle to a boy who had previously been bullying him, but who had not said anything to him in a while and Matti thought was being ‘pretty nice’ to him. However, as he handed over the ladle, the boy thanked Matti, but addressed Matti by his old name, and did so very clearly and slowly for maximum effect. This openly hostile act of misnaming, whereby Matti’s old name was used, and emphasised, served to not only target and hurt Matti, who ‘had chosen not to use that name for a reason’, but also to trigger an emotional reaction in him. Matti stated that he had tried to hide how much it upset him, but that inside he felt ‘very crushed’. Matti’s story reveals the flow of emotions in the school and important sites in the landscape like the cafeteria where emotions might collide. As Matti explained:

That was like my bullies’ biggest thing, being able to poke me by calling me the wrong name, calling me the name that my parents gave me. They took great satisfaction when it came to poking me like that, pushing my buttons, you know. Anything to make me angry or sad.

By referring to being poked and having his buttons pushed, Matti highlights the extent to which misnaming serves as an emotional trigger that can readily be used to negatively affect those who are targeted by bringing about feelings of anger or sadness.

This form of triggering was also raised by 15-year-old Beck, who stated that people would use his friends’ old names ‘to trigger them’. He also pointed to the ways in which the misnaming he had personally experienced had triggered his emotions and left him feeling confused and in pain. Highlighting how such experiences may leave ‘emotional cuts and bruises’ and represent a form of ‘affective violence’ [43] (p. 24), Beck explained that at the time of being interviewed, he had been away from school for approximately 5 months due to what he described as mental health issues connected to his experiences. He had earlier changed lower-secondary school to avoid the bullying to which he was being subjected, only to then experience bullying at his new school. As he explained:

But when I went to the other school, I didn’t know who the people were, but they probably knew who I was, and they went around saying, “Yeah, but Beck was called this before”, and like, “Blah, blah, blah, and stuff like that.” And eventually people passed by me and said, “Yeah, but hey”, my old name then, and said like, yeah, but “she”, “her”, and all that stuff, and it really hurt to hear those kinds of things.

Beck’s story begins to make visible an affective landscape of exclusion. Beck lived in a relatively small town, and despite not knowing the students at his new school, they seemed to know who he was because people had talked about him and his decision to transition and change name. Students at his new school subjected him to both misnaming and misgendering by referring to him as ‘she’ and ‘her’. Beck’s negative experience at the new school was such that he decided to switch back to his original lower-secondary school in the hope of things improving. However, the bullying escalated at his original school, to the extent that he stopped attending lower secondary school altogether.

Beck emphasised that the bullying often focused on his gender identity, with students deliberately dragging up his past through the use of misnaming [23]. Beck gave numerous accounts of the misnaming to which he was subjected, with other students saying things like, ‘Yeah, but Beck isn’t Beck, Beck was this person before’, or repeatedly asking him ‘What’s your name? What’s your name?’ Throughout the interview, Beck emphasised the way in which the misnaming had triggered negative emotions and caused him to cry. He referred to these experiences as ‘really confusing’, ‘pretty difficult’ and as causing ‘real pain’. Not only did the misnaming hurt Beck and cause real pain, but it also served to disorientate him and leave him in a state of confusion due to his place in the world being called into question [30]. That his experiences of misnaming led to him stopping going to school suggests that they were not only points of pressure or stress, but also ‘breaking points’ that pushed him off line and away from the orientation of schooling [31] (p. 248).

Highlighting the ways in which misnaming can function as an emotional trigger for those who are subjected to it, Rowan recalled an example from lower secondary school where he was triggered to such an extent that he had an anxiety attack and began to hyperventilate. Rowan was 16 and had recently started upper secondary school after a difficult time at lower secondary school. He was attending an introductory program to catch up with the subjects he had missed from lower secondary school, because as he put it, he ‘was away a lot during eighth and ninth grade because of psychological reasons.’ Rowan swapped schools in sixth grade because he ‘didn’t really fit into the group’ as ‘the only LGBT person who was open about it’ and began attending a school in a different town instead. However, even at the new school, Rowan was repeatedly subjected to transphobic abuse, including repeated instances of misnaming, whereby students from a parallel class ‘kept saying [his] deadname and shouting at [him] and stuff’ and would ask ‘stupid questions like, “yeah, but what should we call you?”’ Rowan elaborated in detail about a particular incident that began with students from the other class commenting on the colour of his hair, which he had recently dyed, to the point that he went to an area where his friends had their lockers in the hope of having some time to ‘calm [himself] down.’ However, as Rowan explained, the students followed him, and the situation escalated:

And they started calling me my deadname and I just got overwhelmed because there were too many people and I was really anti-social at the time because I really couldn’t take it. And it got to the point where I almost started crying, hyperventilating. I just had a full-blown anxiety attack, and I was sitting there in a corner. I was sitting in the corner under some coat racks with some friends who were standing in front of me to kind of protect me until they left, and then some other people came and wondered what had happened.

While Rowan already needed to calm down after having his hair made the focus of attention, the students’ use of his old name to target him was experienced as a significant stress point that served to disorientate him and trigger an anxiety attack [30]. The anxiety that Rowan experienced restricted his body’s mobility and ability to take up space through a form of ‘shrinkage’ [32] (p. 69), whereby Rowan shrank himself back into the corner, behind the protective legs of his friends. While the commenting on Rowan’s dyed hair can be understood as a straightening device, whereby it was made clear to Rowan that his coloured hair was considered out of place within that peer group, when combined with the misnaming to which he was then subjected, it can be understood as a stopping device, whereby other students sought to challenge Rowan’s queer line and reinforce the cisheteronormative orientation of the school and the peer group.

Jay, who was 18 at the time of being interviewed, gave a number of examples of being misnamed, both at his upper secondary school and online, as well as examples of being challenged about his identity with comments like, ‘what is she doing?’ ‘Yeah, it’s a girl’, and ‘what the hell? You’re not a guy.’ As Jay explained, the misnaming functioned as an emotional trigger that dragged up a past that he had sought to disconnect himself from [23]:

Because [my deadname] is connected to my PTSD so I don’t want to have any connection to it at all, even when my parents and I talk about it. They don’t even say it, and they were the ones who gave me that name. I have such hatred for that name.

Despite not wanting to be connected to his old name anymore, the misnaming to which Jay was subjected served as a stress point that triggered the trauma of his past experiences. Jay explained that he was not only subjected to misnaming by his peers, but also by school staff, and that the misnaming was facilitated by the institutional school cistem that kept his old name alive [23]. Using the analogy of a snapping twig, Ahmed illustrates how a moment of triggering or breaking can obscure the preceding pressure: ‘the snap would only seem the start of something, … if you did not notice the pressure on the twig’ [38] (p. 28). Jay and Rowan were triggered by an event, but in the background of this stress point is an affective landscape of exclusion—the institutional cistem—hammering away and building pressure.

In the following section, we consider more specifically how misnaming may be institutionalised and serve as an administrative straightening device that contributes to the triggering of emotions discussed in this section.

### 4.2. The Institutionalisation of Misnaming as an Administrative Straightening Device

Several of the students interviewed pointed to the importance of changing names at the right time to ensure that their name was registered in the school administrative system and correctly listed on class attendance lists, email addresses, and school equipment like computers. Drew, for example, who was 17, explained that changing his name in the last grade of lower secondary school meant that some school staff continued to address him with the wrong name. However, having changed his name prior to starting upper secondary school meant that he started upper secondary school with the name Drew, which as he expressed, ‘was nice that no one would know my deadname.’ In this sense, the timing of the name change facilitated Drew’s extension into the space of the upper secondary school [30].

In a similar way, Nico, who was 16 and had recently started upper secondary school, said that despite knowing he was trans for a couple of years, he did not come out until his final year of lower secondary school, and did so solely in the hope of having his name changed in the system in time for the start of upper secondary school. As Nico put it:

I only came out in the ninth grade because I hoped I would have time to change my name for upper secondary school so that I could have a new one at the beginning there. I didn’t feel safe coming out at school, but I came out to teachers and friends. Close friends. And then within a day, the rest of the school knew. I didn’t feel safe.

Nico came out to ensure that he could start upper secondary school with a new name and not have to deal with misnaming from peers and school staff. Nico’s impending move to upper secondary school thus acted as a point of pressure that pushed him to come out [30]. However, administrative lag meant that it took 2–4 months for the name change to be registered in the administrative school system. Nico stated that while the class list was adjusted quite quickly, teachers nonetheless used the wrong name when addressing him. This led not only to him being outed in his new school setting but also to an incident of misnaming from a peer, whereby the student shouted his old name at him. While Nico stated that the school social worker was quick to address the incident and stop it from continuing, his sense of insecurity about being out of line in the cisheteronormative school setting meant that he no longer felt able to extend himself into the setting [30]. His sense of insecurity in the school setting meant that he shrank back from the school environment and felt that he ‘was unable to go to the school canteen anymore.’ Nico’s experience highlights the ways in which misnaming contributes to a sense of insecurity and how the fear of being targeted serves to reduce the ability of some bodies to take up space [32]. It also demonstrates how the skin of the social is shaped by the impression the misnaming made, how gendered normativities can linger in a school space, and how Nico’s emotions stuck, not only to the peer who misnamed him, but also to the institutional school setting and the school canteen in particular [30,32].

In a similar way, Eris, who was 19, suggested that even when someone had changed their name prior to changing school, it could still take time for the change to be registered and reflected in the administrative system. Indeed, Eris said that she changed her name when she switched lower secondary schools between eighth and ninth grade, but that she was still allocated an email address with her old name because ‘they hadn’t managed to update the system.’ This example highlights how Eris was stopped from freely extending into (cyber)space through a form of ‘equipmentality’ [30] (p. 46), whereby any emails that she sent or received were addressed to/from her old name. In this sense, the institutional email system was not only a straightening device but also a stopping device that served to stop Eris’ actions and create distance between Eris and her peers [30]. Eris also provided an example of a friend of hers who, despite changing his name prior to moving to upper secondary school, was initially still registered with his old name. As Eris pointed out, such an administrative delay could have serious consequences and lead to the institutionalisation of misnaming, not only within the immediate school setting, but also outside of that setting:

It took him like six months to get his name changed in the system, and then it was really bad, because his old name was on the class lists that went out to the public. His name was on the [online platform] and so on.

Despite having changed his name in time for the transition to upper secondary school, the lag in the administrative system meant that his past was not only dragged back into the present but also thrust into the public domain. While Eris did not elaborate about the consequences this may have had for her friend, it can be assumed from the earlier section that this institutional misnaming may have led to a feeling of disorientation and the triggering of negative emotions connected to this sense of losing one’s place. Disoriented, cut, and bruised, Eris’ friend was required to navigate an emotional landscape of exclusion that included institutionalised straightening devices.

For those who do not manage to change their name in time for a new school start, the risk of misnaming may be even greater. Skylar, for example, talked about a friend of hers who had changed his name one day after starting upper secondary school and was thus registered by his old name in the administrative system and, much like Eris, given ‘an email address with his deadname.’ Jay also stated that he had not managed to change his name in the system prior to starting upper secondary school, and because of this he then had to explain to his teachers that his name is Jay and ask them not to use his old name. While he said that his teachers were generally ‘very good’ at doing so, he had a substitute maths teacher one day who unknowingly read out the students’ names from the class attendance list, where Jay’s old name was still listed:

I also remember this very, very, clearly because she read out all the names from the list, like, and I can understand that, because she can’t know. My teacher maybe forgot to say who I was, which was okay, fine, so I just won’t say I’m here now. So, I asked her to come to me, [and I said] “hey, I’m here but the first name doesn’t match. My name is Jay. My last name is like this. I’m the only one in the whole school. Only my family is called that. Now I’m the only one in upper secondary school whose last name is like this, but this doesn’t match. My name is Jay.”

Despite experiencing his old name as an emotional trigger, Jay described how he understood why the substitute teacher had used his old name while checking the attendance. However, rather than simply accepting what Jay then told her, Jay said that the substitute teacher continued to misname him in front of his peers. As Jay elaborated:

But that she repeatedly said loud and clear in front of the whole class, “yes, but it says deadname, it says deadname, deadname.” And I tried to be quiet about it, because I had just started. I didn’t feel that everyone needed to know.

Jay then pointed at the list on the teacher’s screen and explained that he was listed there but that he did not identify with that name anymore and asked the teacher to refer to him as Jay instead. However, despite pleading with the substitute teacher, he said that she continued to use his old name, until he was triggered to the point of exploding:

Then I exploded. So, then I went out [of the classroom]. And then, after I said some not so nice words to her, I said: “But I told you several times. I pointed at the screen. I said my last name. I explained to you. If you had said it quietly then it would have been OK. I tried to be quiet and calm, but when you continued to push me, and so loudly in front of the whole class, then that’s where the limit is.”

Rather than using Jay’s chosen name, or enquiring more about it in a discrete fashion, the substitute teacher instead orientated herself in line with the class list, while attempting to straighten Jay back into the official line of the institutional setting [30]. Under pressure from the substitute teacher, Jay experienced the misnaming as a breaking point, which not only stopped his extension into the space of the classroom and triggered him emotionally, but also illuminated the inflexible cisheteronormative orientation of the institutional setting. Ahmed uses her analogy of the snapping twig to reveal a consequence of the unseen pressure and hammering prior to the breaking point [38]. Minorities are deemed as the problem, or violent, when they break or snap—‘then I exploded’—when the preceding pressure is not seen. The violence does not begin with the person who snaps but rather originates in the cistem and the affective geographies of exclusion.

A number of the young people called for a more flexible system that is more open to personal preference and provides space for students themselves to indicate which name they would like to be identified as, regardless of whether they have managed to register their change of name in time or not. As Skylar argued:

If you’re a transgender person and haven’t changed your name, then your deadname is listed instead. And [on the application form] you should somehow be able to write that I want this name to be on my application and that this name should appear on my class list.

Skylar points to the need to recognise the ways in which objects like administrative lists affect the skin of the social and the ways in which students are able to orientate themselves and extend into space [30,31]. Likewise, Jay called for school staff to be more willing to listen and talk about identity at school, and for the normalisation of discussions about preferred names and pronoun usage. In other words, Jay highlighted a need for a more balanced ‘co-habitation or sharing of space’ [30] (p. 54) that would allow for an opening up of ‘other paths’, and create new affective landscapes, that are not blocked or restricted by administrative straightening devices [30] (p. 178).

## 5. Discussion

In this study, we explored LGBTQ+ students’ experiences of misnaming in Swedish schools through the lens of orientations and affect. In doing so, we address a lacuna in knowledge about experiences of young LGBTQ+ people in Swedish schools, and particularly in relation to bias-based bullying and misnaming more specifically. Our findings demonstrate how the orientation of schools towards some students more than others determines the extent to which certain students may feel in place or at home while others may experience disorientation and a sense of loss of place [30,31]. As the experiences presented in this study illuminate, cisheteronormative school climates facilitate and encourage the use of straightening and stopping devices, such as the use of mean and derogatory rhetoric and jargon, ridicule, and practices of misgendering and misnaming [30]. Together, these contribute to an undermining of LGBTQ+ young people and their ability to extend into the affective geographies of school spaces.

In line with previous research, our findings suggest that LGBTQ+ students are not only at risk of bias-based bullying, but that transgender and non-binary students are at particular risk of misgendering and misnaming more specifically [4,19,20,21,22,23,24]. Similarly to earlier studies [23,24], our findings also emphasise that such practices negatively affect those who are subjected to them by disorientating them and triggering negative emotions such as anger, sadness, anxiety, and hurt. As Ahmed points out, moments of disorientation are important ‘bodily experiences that throw the world up, or throw the body from its ground’ [30] (p. 157). School bullying, bias-based bullying, and misnaming more specifically, are, as Lohmeyer and Threadgold point out, ‘inescapably emotional’ experiences that leave ‘lasting affective consequences’ [44] (p. 491). As we have shown, misnaming not only leaves an impression on those directly subjected to it, but the affects associated with practices of misnaming also stick and travel and accumulate into collective emotional experiences that shape the social environment of schools [30,39]. They serve to align some people in the space together while creating distance between othered students and students and school staff, and serve to saturate the school context as a site of emotional tension [32].

Our findings also demonstrate how misnaming may be institutionalised within schools and used by school staff, whether inadvertently or deliberately, in their interactions with transgender and non-binary students. Indeed, reinforcing findings from previous research [20,21,24], our findings also point to a lack of awareness about the importance or widespread nature of misnaming and to the ways in which school staff are implicated in the misnaming of transgender and non-binary students [19,24,25]. This points to the cisheteronormative orientations and exclusionary affective geographies within these school settings. At the institutional level, this study highlights the importance of addressing the inadequacies of the administrative system [23,24,25] and working to reorientate schooling to make it inclusive and facilitate the extension into space of LGBTQ+ students so that they can orientate themselves and can feel at home rather than feeling disorientated and out of place [30,31]. At the interpersonal level, making the exclusionary affective geographies and their hammering impact visible provides insight, promotes prevention, and compels compassion for the student who appears to snap. Moreover, this perspective points to the institutional origins of violence.

## 6. Conclusions

Taken together, this study points to the importance of addressing the role of social, institutional, and societal norms in driving the power dynamics that are central to school bullying. By addressing the orientations of schools and the ways in which cisheteronormativity influences the school organisation, its anti-bullying work, and the everyday experiences of young people, it is possible to shift the focus away from the behaviour and effects of bullying towards a more nuanced and complex understanding that takes into account the multitude of factors that may underpin such interactions. Rather than simply addressing negative actions in schools and the behaviour and resilience of individual students, it is necessary to consider the role of social, societal and institutional norms in forming the affective geographies of schools. As the young people in this study have emphasised, practices of misnaming are not only engaged in by individuals, but are also institutionalised through administrative practices that serve to forcefully drag the past of young LGBTQ+ people back into the present despite their desire to move on and extend themselves into schools anew. This points to a need to rethink and reorganise the structure of schools to make them spaces where all students can find their feet, feel at home, and extend themselves in meaningful ways.

## Data Availability

The data presented in this study are available on request from the corresponding author due to ethical concerns.
